# The Effect of Bariatric Surgery upon Diabetes Mellitus: A Proof of Concept by Using the Case of the Mid-Term Effect of Lap Adjustable Gastric Banding (LAGB) on Patients with Diabetes

**DOI:** 10.3390/metabo12121236

**Published:** 2022-12-09

**Authors:** Zvi H. Perry, Uri Netz, Sharon Tzelnick, Ofri Berar, Shahar Atias, Leonid Lantsberg, Eliezer Avinoach, Solly Mizrahi

**Affiliations:** 1 Department of Surgery A, Soroka University Medical Center, Beer-Sheva 84100, Israel; 2 Department of Public Health, Faculty of Health Sciences, Ben-Gurion University of the Negev, Beer-Sheva 84100, Israel

**Keywords:** diabetes, obesity, bariatric surgery and mechanisms, lap gastric banding

## Abstract

Obesity is a worldwide epidemic, with numbers on the rise in the world. Obesity is strongly correlated with increased morbidity and mortality. One of the major factors affecting this increase is comorbid diseases such as diabetes mellitus (DM), which is strongly associated with and dependent on the degree of obesity. Thus, it is not surprising that when efficient surgical treatments were found to battle obesity, researchers soon found them to be relevant and effective for battling DM as well. Laparoscopic Adjustable Gastric Banding (LAGB) is a common surgical treatment for morbid obesity. LAGB has the potential to improve control of the comorbidities of morbid obesity, primarily diabetes mellitus (DM). Our hypothesis was that patients treated with LAGB would have a long-term improvement in the control of DM and that due to its unique mechanism of action, this can lend us a better understanding of how to battle diabetes in an efficient and effective way. This was a cohort study based on patients who underwent LAGB surgery in our institution 4 to 7 years previously and had DM type 2 at the time of surgery. Data were collected from patient’s charts and a telephone interview-based questionnaire including demographics, health status, and quality-of-life assessment (Bariatric Analysis and Reporting Outcome System [BAROS]). Seventy patients participated in the current study. The average follow-up time was 5.1 ± 0.9 years post-surgery. The average weight prior to surgery was 122.0 ± 20.2 kg, and on the day of the interview it was 87.0 ± 17.6 kg (*p* < 0.001). The average body mass index before surgery was 43.8 ± 5.1, and on the day of the interview it was 31.2 ± 4.8 (*p* < 0.001). On the day of the interview, 47.1% of the participants were cured of DM (not receiving treatment, whether dietary or pharmacologic). The sum of ranks for diabetes was lower after the surgery (*p* < 0.001), as was HTN and its treatment (*p* < 0.001). We have shown in this study that LAGB is an effective treatment for morbid obesity, as well as two comorbidities that come with it—DM type 2 and Hypertension (HTN)—in a longer period than previously shown, and with a unique look at the underlying mechanism of action of this procedure. There is a need for further studies to consolidate our findings and characterize which patients are more prone to enjoy these remarkable surgical benefits.

## 1. Introduction

Obesity, defined as a body mass index (BMI) of more than 30, is a worldwide epidemic, with numbers on the rise in the Western world [1,2]. Obesity is strongly correlated with increased morbidity and mortality. One of the major factors affecting this increase is comorbid diseases such as diabetes mellitus (DM), ischemic heart disease (IHD), and arterial hypertension (HTN), which are strongly associated with and dependent on the degree of obesity [3,4]. DM type 2 and HTN are among the most important and dangerous of these [5]. Diabetes was first recognized around 1500 b.c. by the ancient Egyptians, who considered it a rare condition in which a person urinated excessively and lost weight [6]. If you use PubMed, you will find close to 900,000 articles concerning diabetes (1 October 2022). Patients can be asymptomatic but might suffer from Polyuria, Polydipsia, Polyphagia, Fatigue, and Weight loss; at present, most patients are discovered while performing screening [7,8]. The risk of developing DM is strongly affected by familial history and an elevated BMI; together, they nearly double the risk [9,10]. The study of diabetes and related aspects of glucose metabolism has been such fertile ground for scientific inquiry that 10 scientists have received the Nobel Prize for diabetes-related investigations since 1923. One can summarize DM as a chronic condition that is characterized by raised blood glucose levels (Hyperglycemia). Multiple Complex Pathophysiological Abnormalities occur in DM. Plasma glucose is tightly regulated by several hormones like insulin, glucagon, and cortisol. There are a few kinds of diabetes which include Type I (mainly autoimmune in nature) and Type II, which is attributed to a decrease in the response to insulin (like lower glucose uptake in the muscle and fat tissue), or upregulation of glucose production (in the liver). Even though many studies have been conducted, the mechanism of insulin resistance is still unclear, and both genetic and environmental factors seem to be in play. In a cohort study comprising more than 80,000 patients, the strongest predictor for DM after a decade was obesity [11]. In the overall U.S. adult population, the unadjusted prevalence of total diabetes increased from 7.7% in 1999–2000 to 13.3% in 2015–2016 [12]. Thus, it is not surprising to find that there are many different types of treatments for diabetes, in which our aim is to gain near-normal glycemic control to reduce the risk of developing microvascular disease complications [13].

The major known treatments for DM are in three different tiers. First and foremost, the usage of proper dieting and exercise; if that fails, one should revert to oral hypoglycemic therapy, and if that is not enough, insulin therapy might be mandated [14]. Elevated BMI levels have been found to correlate with an overall increase in both morbidity and mortality. The relative risk for diabetes in patients with a BMI > 30 is 93 in females and 42 in males [15]. Thus, it is not surprising that when efficient surgical treatments were found to battle obesity, researchers soon found them to be relevant and effective for battling DM as well [10]. This new approach to treating DM stems from the intimate relationship between DM and obesity, sometimes referred to as the twin epidemics [16]. Bariatric surgery for the treatment of obesity has an important role. It has been shown to be the best long-term solution for weight reduction in the severely or morbidly obese [10,17]. Schauer et al. [18,19] demonstrated that surgical treatment by sleeve gastrectomy or Roux-en-Y gastric bypass on morbidly obese patients with DM was superior to medical treatment in a 1-, 3-, and 5-year follow-up. Bariatric surgery has also been shown to be effective for improving the control of DM, even in patients who have a BMI less than 35 [20,21], and different kinds of bariatric procedures have shown these improvements [22]. The question is: how can surgery treat obesity and thus treat DM?

The mechanism by which weight loss surgery improves weight are divided into three main mechanisms [23]:

- Modifications of the enteroinsular axis—Malabsorptive;
- Reduce certain GI hormonal level;
- Reduce food intake—Restrictive.

The hormonal effect of bariatric surgery has been extensively studied, such as in studies by Dimitriades et al., Pg Baharuddin [24,25]. Understanding the mechanisms mediating the weight loss and metabolic effects of bariatric surgery is key for developing less invasive procedures and medical obesity treatments. Post-operative changes in circulating gut hormones, including ghrelin, peptide YY (PYY) and glucagon-like peptide-1 (GLP-1), are thought to play a key role to the beneficial outcomes of bariatric surgery, as seen in myriad bariatric procedures like Lap Sleeve Gastrectomy (LSG) [26]. The malabsorptive mechanism of action is unique only to specific procedures like Roux-N-Y Gastric bypass, Duodenal Switch and One anastomosis gastric bypass [27]. Bariatric procedures are divided into three main mechanisms of action: Restrictive, Malabsorptive and a combined mechanism. Many of the surgical procedures involve more than one of these mechanisms, such as Roux-N-Y gastric bypass (RYGB), which involves both a restrictive component (creating a small gastric pouch) and a malabsorptive component (bypassing a certain length of the small bowel). Biliopancreatic diversion (BPD) is mainly a malabsorptive procedure (bypassing most of the small bowel) but it still has a restrictive part to it (creating a sleeve to the end of which the small bowel will be anastomosed). Even procedures thought to be mainly restrictive procedures like Lap sleeve gastrectomy (LSG) have a specific hormonal effect due to cutting to gastric fundus, which is the main driver for ghrelin secretion. Thus, we are unable to deduce which of these components is key to treating diabetes. This led us to look for a purely restrictive procedure with no other components of action such as a direct hormonal effect, anatomical change or a malabsorptive effect. This led us to the current study research question: what if the effect of bariatric procedures on diabetes is mainly restrictive?

We need a surgical procedure that is with no known (or even suspected) hormonal or malabsorptive mechanism of action. Fortunately, this is the case with a procedure termed Lap Adjustable Gastric Banding (LAGB)—a minimally invasive bariatric procedure which is restrictive in nature. One can change the size of the stomach through a port that is situated in a sub-cutaneous access point. The worldwide utilization of LAGB as a bariatric procedure has varied, with it being used in 24.4% of all bariatric procedures in 2003, peaking at 42.3% in 2008, and decreasing to 17.8% in 2011 [28]; it now comprises less than 5% of bariatric procedures done in the USA [29]. However, its limitations have made the lap band less popular. First and foremost, there are long-term complications with the band (slippage, erosion and intolerance, to name a few [30], which has led to a high rate of band removal [31]. Another issue is the fact that the lap band needs frequent adjustments in the clinic, which is not compensated fully by insurers, thus putting a financial strain both upon the physician and patient (thus these patients are called sometimes PFLs—patients for life). Last but not least is the fact that there are newer and more efficient surgical procedures like lap sleeve gastrectomy (LSG), lap gastric bypass (RYGB), etc. (as seen in the study of Ding et al. [32] which has shown superior results regarding diabetes remission in LSG and RYGB). Thus, with time the usage of LAGB has declined even though it is considered very safe, until these days in which LAGB comprises less than 5% of bariatric procedures performed worldwide. Still, the case of the gastric band is a good example of a pure restrictive procedure—no physiological changes or resections. The band is placed laparoscopically around the upper stomach to create a 15 mL pouch. A port connected to the band is extracted to the abdominal wall and placed beneath the skin. Inflation of the band is done via the port in a clinic scenario, and weight loss can be up to 50% of Excess Body Weight (EBW).

It is a relatively simple and safe operation that does not change gastrointestinal anatomy (allowing reversibility), has a very low mortality rate and short hospital stay, and results in good, stable long-term weight loss [31,33]. Thus, LAGB is the best way to study the pure effect of restriction upon obesity at large and specifically in relation to Diabetes Mellitus. In this study we aimed at examining the long-term effect of LAGB on DM and HTN. Our hypothesis was that LAGB would be proven to be an effective treatment for DM and HTN in a long-term follow-up 4 to 7 years after the index operation.

## 2. Materials and Methods

This was a retrospective cohort study based on patients with obesity and DM that underwent LAGB. All surgeries were performed at the Soroka University Medical Center (SUMC), a high-volume bariatric center situated in southern Israel. The study was approved by the local Institutional Review Board (SOR-11-0049). Inclusion criteria included patients who underwent LAGB (ICD-9 code 44.95) in the department of surgery A during the period 1 January 2002 to 31 December 2012, had a concomitant diagnosis of DM type 2 prior to surgery, and a follow-up period of 4 to 7 years post-surgery.

Exclusion criteria were patients who were lost to follow-up, patients who did not consent to participate in our study, and patients who were suffering from a concomitant grave disease (like cancer, heart failure, etc.). From the cohort of patients fitting these criteria, a random sample of 70 patients was selected to be included in the study. Consenting participants were interviewed by telephone.

Comorbidities and weight loss were assessed by a standardized questionnaire (see Supplement S1) [30]. Outcomes were assessed according to the Bariatric Analysis and Reporting Outcome System (BAROS) questionnaire, which is a standardized questionnaire for assessing weight loss and quality of life in bariatric surgical patients [34,35] (Supplement S2). Additional data were collected from patient charts.

### 2.1. Surgical Technique of LAGB 

The LAGB procedure was performed as previously described [30]. To aid those who are less familiar with this procedure we have added in Appendix A, in full length, the surgical technique and figures to illustrate the band placement and different band types.

### 2.2. Power Analysis

The power of the study was calculated using PEPI-for-Windows (WINPEPI) (Abramson PJ. WINPEPI (PEPI-for-Windows). 2016; Available online: http://www.brixtonhealth.com/pepi4windows.html (accessed on 15 January 2017; updated 23 August 2016) by comparing the total BAROS score before and after surgery (pairs function). We used a power of 80%, α = 0.05, a difference of at least 1 in the total BAROS score, with a standard deviation of 2 (as seen in prior studies of ours like Lewis et al). Under these assumptions, the required sample size was at least 51 pairs of patients for a 2-sided test.

### 2.3. Statistical Analysis

The data were analyzed with SPSS version 25.0 (SPSS, Chicago, IL, USA) software. Descriptive statistics including mean values and standard deviations were used to describe the baseline characteristics of the 2 study groups. Intergroup comparison was done using the Pearson chi-square test for qualitative variables and the Fisher exact test for dichotomous variables. Correlations were measured using Pearson’s correlation for parametric variables, and the Spearman correlation was used for variables whose distribution defied the assumptions underlying the normal distribution.

Comparison of quantitative variables was performed using parametric Student’s *t*-test for paired samples and non-parametric Wilcoxon test. The level of significance was defined as *p* < 0.05.

## 3. Results

**Figure 1:**
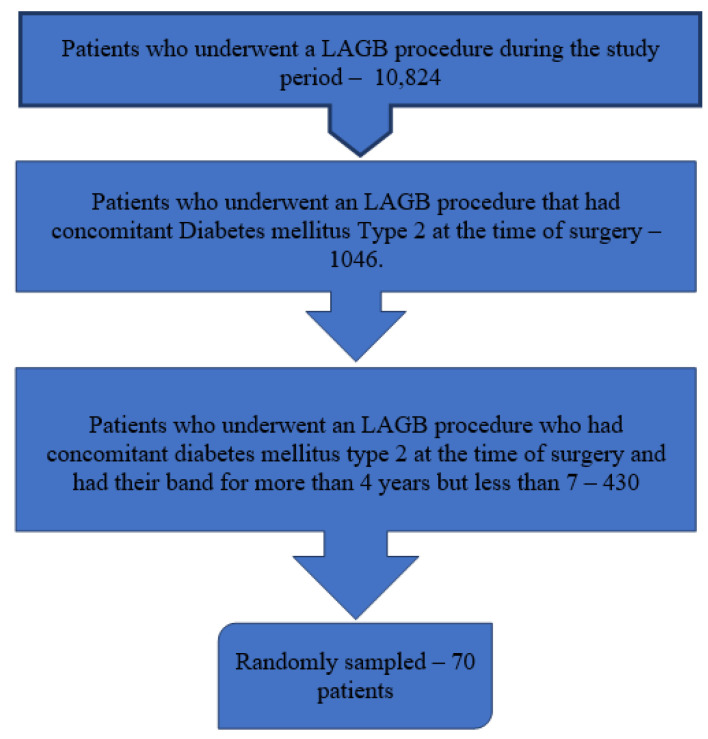
Study Population. *LAGB*, laparoscopic adjustable gastric banding.

A total of 70 patients who fit the above criteria were enrolled. Figure 1 depicts the flow diagram for patient selection. Twenty-three (33%) patients were male, and 53 (76%) of the patients were married. The mean age at recruitment was 55 ± 9.9 years. The average time after surgery was 5.1 ± 0.9 years. There were no early complications in the index hospitalization. In 10 patients (14%), the band was empty (open) during the time of the interview (an empty band does not contribute to weight loss as it does not compress the stomach). The average weight prior to surgery was 122.0 ± 20.0 kg; the lowest weight attained was 78.0 ± 16.4 kg, while the average weight at the time of the interview was 87.0 ± 17.5 kg. The average weight loss between surgery and the interview was 35.0 ± 16.6 kg (*p* < 0.001). A summary of this is depicted in Table 1.

The average BMI immediately prior to the operation was 43.8 ± 5.0 kg/m^2^, whereas at the time of the interview the average BMI was 31.2 ± 4.8 kg/m^2^, with an average decrease of 12.6 ± 5.7 kg/m^2^ (*p* < 0.001). No significant differences were found in weight loss or BMI between males and females (*p* = 0.946).

**Figure 2:**
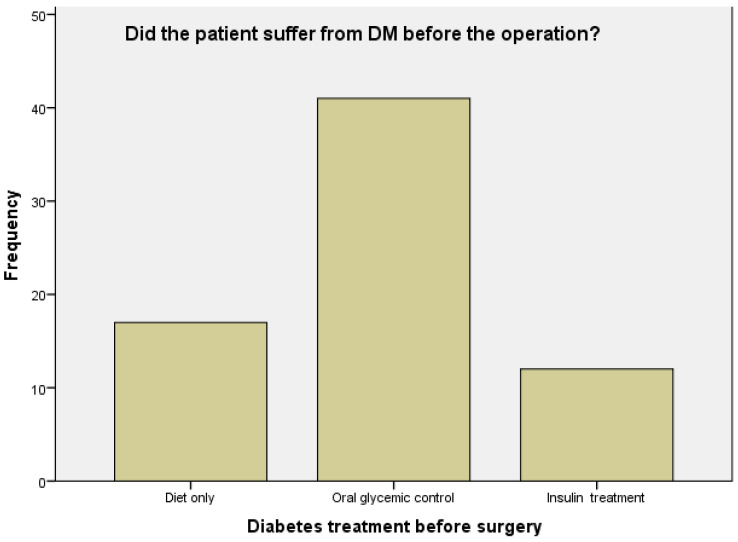
Diabetes mellitus (DM) status prior to the operation. Conventional treatment means weight management and diet only.

**Figure 3:**
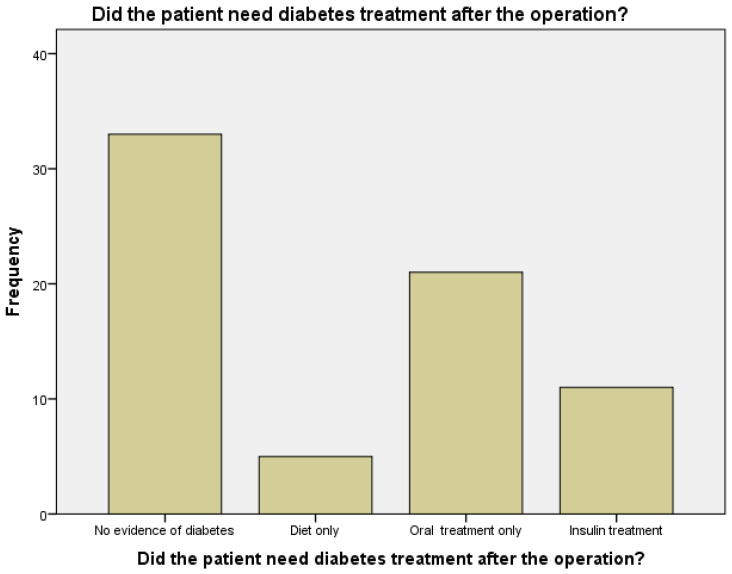
Diabetes mellitus (DM) status after the operation.

As mentioned, all patients had DM prior to the operation; 17 (24%) were treated by diet only, 41 (59%) needed oral medications, and 12 (17%) needed insulin (see Figure 2). After the operation, 33 (47%) of the patients had a remission from diabetes, 5 patients (7%) needed only diet as their treatment, 21 patients (30%) needed oral medications, and 11 patients (16%) needed insulin (*p* < 0.001, see Figure 3). When patients were asked about their diabetes state, only one did not answer this question, 3 patients (4%) said that they felt it had gotten worse, 8 (11%) declared no change in disease status, 8 (11%) declared a slight change in disease status as being more balanced, 18 (26%) reported a big improvement in their status, and the rest (32 patients, or 46%) reported remission in their diabetes. When patients were asked about their use of diabetes and HTN medication before and after the operation, their responses showed a significant decline (*p* < 0.001). The average fasting glucose level of 47 patients who were tested in the month of the interview was 111.6 ± 32.0. Forty-three patients (61%) had HbA1c levels taken before and after the operation—of these, the average HbA1c before the operation was 8.6 ± 2.1%, and after the operation the average was 6.6 ± 1.1%; this led to an average decrease of HbA1c of 2.1 ± 2.1%, which was a significant decrease in the HbA1c from before the operation (*p* < 0.001).

Of the 70 patients, 39% had lost less than 25% of their excess body weight, while other patients lost more than 25% of their excess body weight. When looking at the group that had lost more than 25% of their excess body weight, there was a higher utilization of the band (95% vs. 70% in the lower reduction of band utilization; *p* = 0.011). Age and number of years with the band had no significant effect on excess weight loss. An interesting result was that there was no difference between those who lost more than 25% of their excess weight and those who lost less than that in the reduction of HbA1c, which means this is unrelated to the magnitude of the weight loss. Also, we did not find any correlation between the HbA1c levels and the BMI decrease or the excess weight loss (no significant correlation was found between the HbA1c levels and delta BMI [r = −0.105, *p* = 0.5], as well as total weight loss [r = −0.083, *p* = 0.6]). Another interesting result was an improvement in other comorbidities such as HTN (50% did not need any HTN drugs 5 years after the operation) and Obstructive Sleep Apnea (OSA), which has clinically improved in 25% of the patients. When considering the subjective level, 29% of the patients felt a significant improvement in their self-esteem, and 43% felt a dramatic improvement. Forty-nine percent of the patients felt a significant improvement in their physical ability, and a significant correlation was found between the excess weight loss and the improvement in physical ability (*r* = 0.421, *p* < 0.001). When considering weight in the interview, we found an inverse correlation between the current weight and the BAROS score (*r* = −0.385, *p* = 0.001). A similar result was found between the BMI difference and the BAROS score (*r* = 0.604, *p* < 0.001). Similarly, a significant difference was found in the total BAROS score between the group who lost less than 25% of their excess body weight and those who lost more (1.6 vs. 3.3; *p* < 0.001). A summary of the quality-of-life instances is depicted in Table 2.

## 4. Discussion

Obesity and its comorbidities, mainly diabetes [13] and hypertension, are a great challenge to physicians today [3]. Surgical therapy for obesity and its comorbidities is well established [20,21,23,30]. Research has shown bariatric surgery’s efficacy and safety for treating obesity-related illnesses [29,31,36], mainly diabetes [37,38]. Numerous recent randomized clinical trials directly comparing various surgical vs non-surgical interventions for diabetes [37,38] uniformly demonstrate the former to be superior for improvements in all glycemic variables and other metabolic endpoints [19,39,40,41].

However, these studies were for short periods of time, and their long-term efficacy is not well established; moreover, the mechanism of action upon diabetes is still unclear. This led us to conduct the current study. In our study, we followed patients with diabetes who had undergone LAGB for a range of 4 to 7 years after surgery, with a mean follow-up time of 5+ years. When considering the long-term effect of LAGB on obesity, our study has shown that 5 years after the operation our patients showed a stable and significant decrease in body weight (*p* < 0.001); the mean BMI prior to the operation was 43.8 ± 5.1 kg/m^2^, and the mean BMI at the time of the study was 31.2 ± 4.8 kg/m^2^, which reflects a mean BMI reduction of 12.5 kg/m^2^, or 35 kg lost on average. Sixty-two percent of the patients lost more than 25% of their excess body weight. Similar results have been demonstrated in other studies, even though their follow-up period was shorter [42,43] and there was a lack of clarity regarding the mechanism of action of surgery upon diabetes [38,41]. Our study is mainly used here as a way to elucidate the mechanism in which bariatric surgery might aid the treatment of patients with diabetes. Understanding the mechanisms mediating the weight loss and metabolic effects of bariatric surgery is key for developing less invasive procedures and medical obesity treatments [44,45,46]. There are at least three main theories that try to explain the effect of bariatric surgery upon obesity: One theory talks about modifications of the entero-insular axis—this is mainly attributed to malabsorptive procedures like Roux-N-Y gastric bypass (RYGB) [44,47,48]. Thus for example, Salehi et al. [48] found that post-RYGB, islet hormone secretion is altered as a result of factors beyond circulatory glucose levels. If this explanation was the main effect of bariatric procedures, then other surgical procedures that do not alter the entero-insular axis would not have been as successful in treating diabetes. However, research has shown that restrictive procedures like sleeve gastrectomy are as efficient in the mid-term treatment of diabetes [26,49]. Thus, another explanation was that surgery reduces certain GI hormonal levels, as is the case with lap sleeve gastrectomy (LSG). In general, weight loss and improved glycemic control after LSG have been explained by another theory denominated gastric hypothesis, which delineates changes in the secretion of gastric factors triggered by direct manipulation of the stomach as responsible for the rapid restore of insulin and increased sensitivity to it. The main peptide involved in this situation is ghrelin, produced by the gastric fundus, a tissue excised during LSG [27].

Ghrelin is a peptide hormone produced by ghrelinergic cells in the gastrointestinal tract and functions as a neuropeptide in the central nervous system. Besides regulating appetite, ghrelin also plays a significant role in regulating the distribution and rate of use of energy [50,51]. Ghrelin is mainly known for promoting appetite increase in humans and rodents. Its decrease seems to accelerate the gastric emptying and the intestinal transit by the fast delivery of nutrients to the duodenum and the large intestine, thus influencing gut hormones such as GLP-1, peptide YY, GIP, and leptin34. There is also evidence that ghrelin acts over the β-cell as well the body weight [52]. However, if this mechanism was true, or the main explanation for the beneficial effect of surgery on Diabetes, then a pure restrictive procedure like lap gastric band would have shown no short- or mid-term effect on the diabetes status of the patients. The lap gastric band involves no physiological changes or resections and no anatomical alterations. However, as our study has shown, forty-seven percent of our patients, who at the start of our study were all diagnosed as patients suffering from diabetes, no longer had elevated blood sugar, which meant that they needed no treatment. HbA1c levels decreased from 8.6% to 6.6%, which means that on average, most of our patients have become balanced regarding their diabetes status. Our results have validated and shown that the remission rate stays higher 5 or more years after the procedure. This means that the basic mechanism that has aided our patients was pure restriction and the reduction in food intake per se. Our results are echoed in a larger study, the Swedish Obese study [53], in which 1658 patients who underwent bariatric surgery and 1771 obese patients matched controls. Bariatric surgery appears to be markedly more efficient than usual care in the prevention of type 2 diabetes in obese persons, when many of the patients studied had pure restrictive procedures like gastric banding or vertical banded gastroplasty. This was also seen in a study where randomized patients, either through traditional diabetes treatment or LAGB, showed that remission rates were five times more likely in LAGB patients 2 years after the operation [43]. Our results have validated that and have shown that the remission rate stays higher 5 or more years after the operation. If we take into consideration the long-term damage caused by diabetes and the dangers of traditional treatment for it (mainly hypoglycemia), the LAGB patients on average have shown a normal HbA1c, which means a balanced sugar level not only on the day of the examination but at least 3 months prior to it; this means that the operation has succeeded in the long-term control and even the “cure” of their diabetes.

If we remember that one of the indications for bariatric surgery is alleviating comorbid illness, mainly diabetes, our results show that long-term relief is achieved using LAGB and that this remission is independent of the excess body-weight loss, which mitigates one of the main criticisms about LAGB, that it leads to a smaller reduction of weight than other bariatric procedures.

When considering subjective results of the LAGB, we have found (not surprisingly) that the larger reduction in weight (as portrayed in excess weight loss) was positively correlated with the elevation of self-esteem after the operation. Previous studies have shown that 1 year after surgery, the weight loss was not a predictor for the quality of life of bariatric patients [54,55], and we can understand from this that the change in weight loss is significant only on larger time scales and that the main factor is not weight by itself but the loss of excess weight. We have seen that 5 years after the operation, the total BAROS score was on average 2.7, which is considered a fair result. This seems to be contradictory to the fact that remission and a clear improvement in diabetes are seen in most of our patients. We believe this stems from the fact that even though bariatric operations are lifesaving procedures in essence, they are perceived by the general population as an aesthetic procedure, and due to that, the satisfaction from the operation is reliant mainly on weight loss and not on true objective measures such as HbA1c. Thus, it seems logical to try to coordinate the patients’ expectations to aid them in their subjective evaluation of the operation.

### Study Limitations

First and foremost, the sample size is not very large, even if it is satisfactory based on sample-size calculations. Second, our study is in part a retrospective cohort study, which is a design more prone to biases such as information and selection bias. It would be better if this study had been conducted in a prospective cohort study design, but time and financial constraints prevented this. Another limitation is the length and timing of the study. We did not see, in the years of the study, any trend of more band removals, but in the last 2 years, as the sleeve has become increasingly prevalent in Israel, we have seen a rise in the number of band removals. Based on these limitations, we advise that future studies should have a larger sample size, should include patients whose band has been removed, and should involve longer periods of follow-up.

## 5. Conclusions

Our study is unique in its length of follow-up on patients with diabetes who underwent LAGB. We have found that LAGB is an efficient and safe procedure not only for weight loss, but more importantly for the comorbid illnesses that are so prevalent among obese patients—mainly diabetes and HTN. Our study has shown that 5 years after the operation, half of our patients were in remission of their diabetes and that most of the patients showed an improvement in their diabetes, in sharp contrast to patients with diabetes who were not operated on, a majority of whom showed deterioration in their medical status. Considering the unique mechanism involved in LAGB, we can conclude that diabetes can resolve by the restrictive mechanism per se and not due to hormonal changes or malabsorptive changes seen in other surgical procedures.

Thus, we can conclude that in the long term, LAGB at least ceases the detrimental deterioration seen in diabetes, and it might even lead to a full remission of the disease.

Since the public burden of diabetes worldwide is enormous, and today treatments for this disease are lacking, LAGB might be a valid answer, both safe and efficient, and a big advancement in our understanding of how to combat diabetes.

## Figures and Tables

**Table 1:** Pre- and post-op comparisons.

Variable	Pre-Op	Upon Interview	*p*
Average weight, kg ± SD	122.01 ± 20.00	86.97 ± 17.53	˂0.001
Minimal weight attained	122.01 ± 20.00	78.68 ± 16.35	˂0.001
BMI—kg/m^2^, mean ± SD kg/m^2^	43.76 ± 5.08	31.16 ± 4.83	˂0.001
Minimal BMI attained kg/m^2^	43.76 ± 5.08	28.20 ± 4.53	˂0.001
HbA1c % ± SD	8.60 ± 2.10	6.6 ± 01.10	˂0.001
The median level of diabetes drugs usage (range 1–4)	2	1	˂0.001
The median level of HTN drugs usage (range 1–4)	2	0	0.001

**Table 2:** The post-op quality of life parameters.

Parameter	Central Tendency Value
Self-esteem—Median (Range)	4.0 (2.0)
Physical activity—Median (Range)	3.0 (3.0)
Social function—Median (Range)	3.0 (3.0)
Function at work—Median (Range)	3.0 (3.0)
Sexual function—Median (Range)	3.0 (3.0)
BAROS summary index for quality of life—Mean (s.d.)	0.83 (0.77)
BAROS summary index for weight loss—Mean (s.d.)	0.64 (0.54)
BAROS summary index for medical improvement—Mean (s.d.)	1.2 (0.88)
Total BAROS score—Mean (s.d.)	2.67 (1.43)

## Data Availability

The data presented in this study are available in the main article and the Supplementary Materials.

## Supplementary Materials

The following supporting information can be downloaded at: https://www.mdpi.com/article/10.3390/metabo12121236/s1, Figure S1: Lap band placement around the stomach, Figure S2. Different types of Lap Gastric Band, Figure S3. The lap band ports’ positions: Insertion sites of the 10-mm trocar sleeves (I, II, III) and the 5-mm trocar sleeves (IV, V); Supplement S1; Surgical Results and Comorbidity Questionnaire, Supplement S2; Bariatric Analysis and Reporting Outcome System (BAROS) obesity scale.

## Appendix A. Gastric Band Surgical Technique

The patient is placed on the table in the supine position with left tilt. All patients were administered a single dose of intravenous cefuroxime (750 mg) preoperatively unless there was a known allergy to it, and if that was the case, they received 600 mg of IV clindamycin. End-tidal CO_2_ monitoring was performed in all patients during surgery. The pneumoperitoneum was achieved using Veress needle in all patients. The Veress needle was inserted in the left subcostal space in the middle clavicular line to avoid inadvertent injury of the uterus. Pneumoperitoneum of 12 mm Hg was obtained. The location of the first port is supra-umbilical and was moved cephalad according to prior operations. Subsequently, two working 10 mm trocars were placed under laparoscopic guidance, as well as two 5 mm ports to aid with retraction (see Figure 3). Standard laparoscopic band technique includes retro-gastric transit of the band: by pulling downward on the gastric fundus with an atraumatic grasper and retracting the left liver lobe upward using a second 5-mm locked grasper, you expose a triangular area in which the three sides in clockwise sequence are the diaphragm, the gastro-phrenic ligament, and the esophagus.

Using the “Goldfinger” instrument (Obtech Medical AG, Baar, Switzerland) one bluntly dissects into the center of the triangle, exposing the angle of His and the retro-gastric pad of fat. Then, one must expose the right aortic crura by detaching the pars flacida. Then, you introduce the Goldfinger, curving its tip into the retro-gastric fat tissue adjacent to the left margin of the crura and directing it toward the exposed angle of His. At this point, one unwraps the sterile band at the back table, fully deflates its balloon, and grasps the band at its end tag, inserting it through the left subcostal opening, snugging the loop into the slit on the Goldfinger tip, and pulling it through the retro-gastric extraperitoneal path. One locks the band-end (see Figure 1). Then, one exteriorizes the injecting tube through the subxiphoid port. The injecting tube is connected to the tube and is inserted in a pre-sternal tunnel. Our current technique does not include the use of a nasogastric tube (except for cases where we look for leaks by applying a methylene blue dye test), a calibration balloon, or high-energy instruments. For different kinds of bands, see Figure 2.

Regarding revisional procedures (i.e LAGB post prior bariatric procedures), adhesiolysis is performed as needed, and dependent upon the previous bariatric procedure, measures are taken—surprisingly, post- sleeve band is easy as there are not a lot of adhesions, and the sleeve itself is smaller and easier to encircle and put a band around it, thus reinforcing the restrictive nature of the LSG. In RYGB, sometimes we saw more adhesions, but the operation is performed around the small gastric pouch, which in most cases accessible, and thus putting the band around it is simple, adding a restrictive portion to it. Our experience with SRVG has shown us that this is slightly more complex as we have seen many adhesions in the surgical area, and one thing we have learned is not to insist on finding the silastic ring itself, but as the LAGB is inserted in a much more proximal location, all you need is to have a proper area retro-gastric to have the Goldfinger inserted and bring it out so that you can engulf the stomach. In the last few years, mainly to prevent slippage of the band, we have added a small tunnel in the adhesions themselves, as an anchor to the band itself.

**Post-op follow-up:** Success in maintaining weight loss after bariatric surgery requires the ability to implement long-term changes in eating habits and lifestyle. Appropriate dietary and lifestyle counseling is essential following bariatric procedures to ensure appropriate macronutrient and micronutrient status.

Thus, we have a set of five clinics nationwide, which gives us a good coverage of most of Israel (which is smaller in size than Rhode Island). In the clinic, as much as 250 patients are seen weekly and can be divided into three major groups:

(a) New patients (~5%) referred for initial evaluation before determining the need for a band surgery;
(b) Patients during the first 6 months after surgery (~60%). These patients are seen monthly in the clinic for inflation of the band to an average amount of 8 cc of normal saline;
(c) Patients 6 months to 10 years after surgery (~35%), coming to the clinic for various reasons such as eating difficulties, vomiting, desire to lose more weight, consultation about proper weight loss expectations and eating habits, etc.

Although general guidelines exist, individual monitoring and tailoring are frequently required, and in the clinics we try to find the best-fit closure of the band, with the necessary medical and nutritional follow-up.
