# Supplementary material for: The Effect of Bariatric Surgery upon Diabetes Mellitus: A Proof of Concept by Using the Case of the Mid-Term Effect of Lap Adjustable Gastric Banding (LAGB) on Patients with Diabetes
Source: Metabolites. 2022 Dec 9;12(12):1236. doi: 10.3390/metabo12121236 (PMC9781846; doi:10.3390/metabo12121236)
Supplement: Supplementary file 1 [file metabolites-12-01236-s001.zip › metabolites-2046601-supplementary.pdf]

Index Figures and supplements

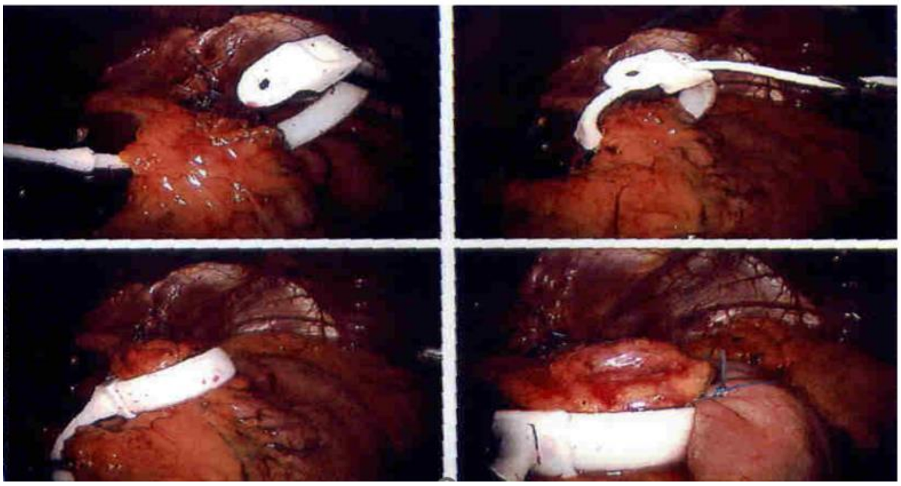

Figure S1. Lap band placement around the stomach

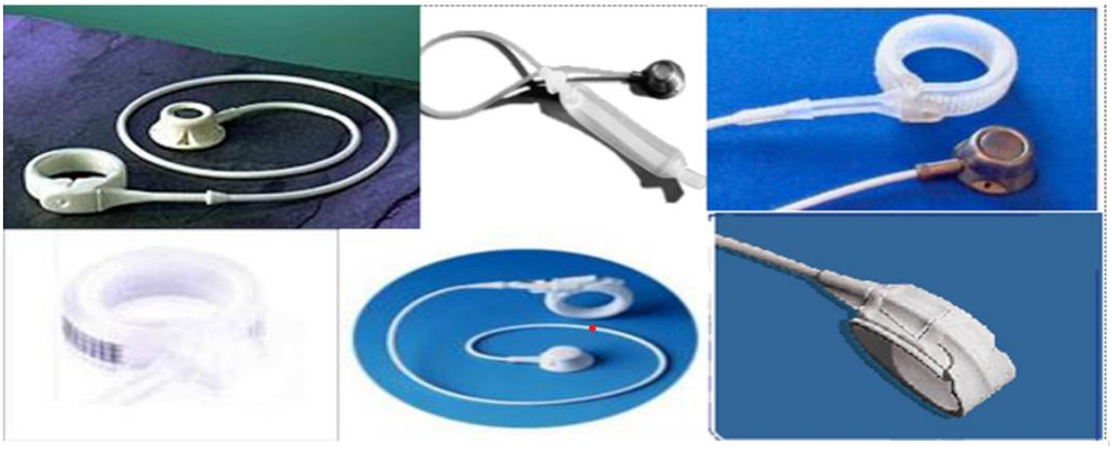

Figure S2. Different types of Lap Gastric Band

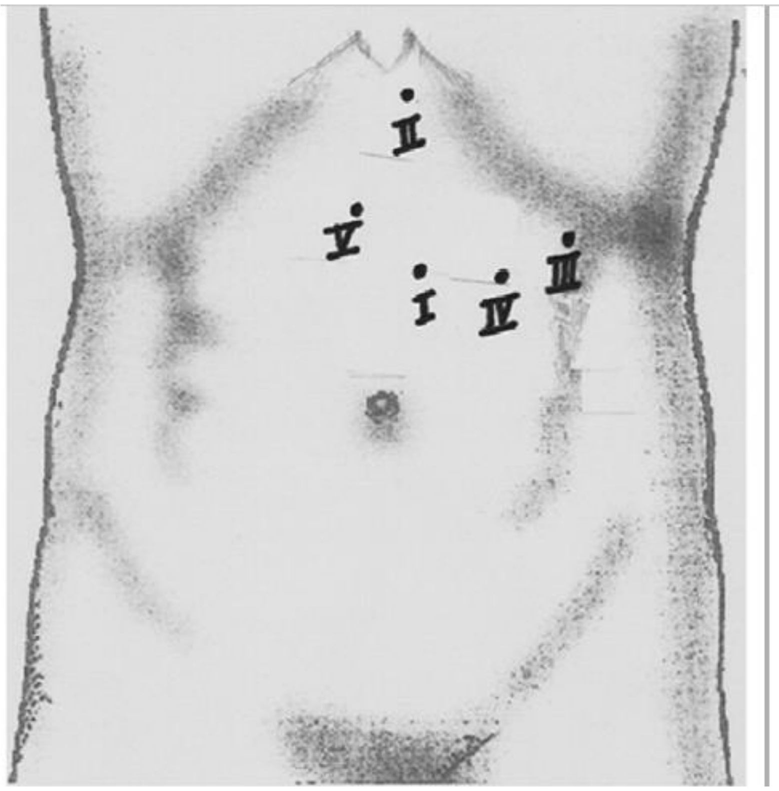

Figure S3. The lap band ports' positions: Insertion sites of the 10-mm trocar sleeves (I, II, III) and the 5-mm trocar sleeves (IV, V).

Supplement S1 – Surgical Results and Comorbidity Questionnaire

Subject number: _____________ Name and Family: _____________

Date of birth: _____________ Gender: M\F

Height (cm): _____________ Current weight (kg): _____________

Lowest weight attained (kg): ________ Year of band placement: _____________ Post-op hospital stay (days):_____________

Were there any early post-op complications? _____________

Any late post-op complications?_____________

Background illnesses:

Did you suffer from HTN prior to surgery?_____________

Do you suffer today from HTN? _____________

How did the surgery affect your HTN? It worsened/Did not change/Some improvement/A big improvement/Total remission

Did you suffer from DM prior to surgery?_____________

Do you suffer from DM today? _____________

How did the surgery affect your DM? It worsened/Did not change/Some improvement/A big improvement/Total remission

Did you suffer from OSA prior to surgery?_____________

Do you suffer from OSA today? _____________

How did the surgery affect your OSA? It worsened/Did not change/Some improvement/A big improvement/Total remission

** DM—Diabetes mellitus, HTN—Hypertension, OSA—Obstructive sleep apnea

Supplement S2 – Bariatric Analysis and Reporting Outcome System (BAROS) obesity scale

WEIGHT LOSS % OF EXCESS (points)	MEDICAL CONDITIONS (points)	QUALITY OF LIFE QUESTIONNAIRE
Weight gain (-1)	Aggravated (-1)	1. SELF ESTEEM: -1.0, -.50, 0, +.50, +1.0
0 - 24 (0)	Unchanged (0)	2. PHYSICAL: -.50, -.25, 0, +.25, +.50
25 - 49 (1)	Improved (1)	3. SOCIAL: -.50, -.25, 0, +.25, +.50
50 - 74 (2)	One major resolved Others improved (2)	4. LABOR: -.50, -.25, 0, +.25, +.50
75 -100 (3)	All major resolved Others improved (3)	5. SEXUAL: -.50, -.25, 0, +.25, +.50
Subtotal:	Subtotal:	Subtotal:

COMPLICATIONS
Minor: Deduct 0.2 point
Major: Deduct 1 point

REOPERATION
Deduct 1 point

TOTAL SCORE

OUTCOME GROUPS SCORING KEY

FAILURE	1 point or less
FAIR	> 1 to 3 points
GOOD	> 3 to 5 points
VERY GOOD	> 5 to 7 points
EXCELLENT	> 7 to 9 points
